# Regulating the BCL2 Family to Improve Sensitivity to Microtubule Targeting Agents

**DOI:** 10.3390/cells8040346

**Published:** 2019-04-12

**Authors:** Robert H. Whitaker, William J. Placzek

**Affiliations:** Department of Biochemistry and Molecular Genetics, University of Alabama at Birmingham, Birmingham, AL 35294, USA; rhwhitaker@uab.edu

**Keywords:** apoptosis, cell cycle, chemotherapy, microtubule, bcl2 family, mitosis, microtubule targeting agents, tubulin, cellular stress

## Abstract

Chemotherapeutic targeting of microtubules has been the standard of care in treating a variety of malignancies for decades. During mitosis, increased microtubule dynamics are necessary for mitotic spindle formation and successful chromosomal segregation. Microtubule targeting agents (MTAs) disrupt the dynamics necessary for successful spindle assembly and trigger programmed cell death (apoptosis). As the critical regulators of apoptosis, anti-apoptotic BCL2 family members are often amplified during carcinogenesis that can result in MTA resistance. This review outlines how BCL2 family regulation is positioned within the context of MTA treatment and explores the potential of combination therapy of MTAs with emerging BCL2 family inhibitors.

## 1. Introduction

Chemotherapeutics aim to exploit unique metabolic differences between cancer and normal cells in an effort to kill cancer cells more rapidly than the surrounding healthy tissue. For this reason, microtubule targeting agents (MTAs, also called microtubule poisons) have been employed in treating a variety of malignancies, including hematologic and solid tumors for decades [1]. MTAs are effective, as they target the persistent growth phenotype exhibited by cancer. This heightened growth potential is one of the key differences between malignant and healthy cells that allows MTAs to be a standard of care in the treatment of multiple human cancers. Despite the prevalent historical and current application of MTAs, an inherent issue of the persistent use of broad chemotherapeutics is the emergence of drug resistant cellular populations. Multiple mechanisms for resistance to chemotherapeutics have been observed in human cancers, including altered drug metabolism, altered clearance, as well as modulation of cell death [2]. For MTAs, a primary determinant of response and resistance occurs through modulation of the stress-induced BCL2 protein family of programmed cell death (apoptosis) regulators. However, microtubules and the BCL2 family are connected through more than just chemoresistance, as both are regulated differentially and sequentially throughout the cell cycle. Indeed, MTAs are often termed “anti-mitotic” due to their significant effect during mitosis, even though microtubule dynamics are involved in processes throughout the cell cycle [3]. The purpose of this review is to outline how BCL2 family regulation is positioned within the context of MTA treatment and the potential that the application of combining MTAs with emerging BCL2 family inhibitors has on improving anti-cancer therapy.

## 2. Microtubules and Tubulins

**Figure 1:**
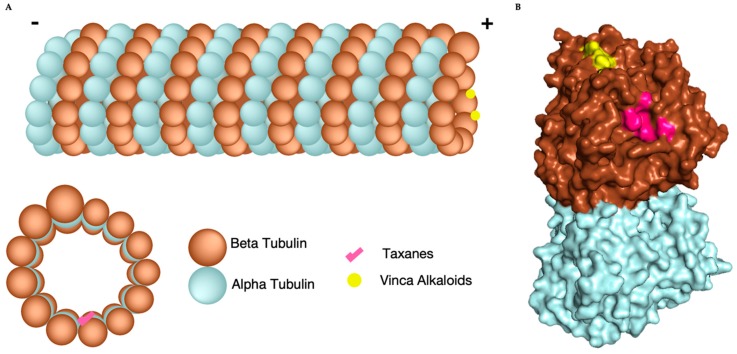
Microtubule composition, structure, and microtubule targeting agents (MTA) binding sites. (**A**) Cartoon of microtubule consisting of 13 protofilaments formed from alpha (blue) and beta (brown) tubulin heterodimers. Vinca alkaloids (yellow) bind to the + end of the microtubule and taxanes (pink) bind inside the lumen (lower left). (**B**) Space Filling model an of alpha (Blue) beta (Brown) heterodimer with vinca alkaloid binding site (yellow) and taxane binding site (pink) located on beta tubulin [PDB: 3J8Y].

Microtubules are polymeric protein assemblies that are involved in many diverse cellular structures and processes. They have key functions outside the cell cycle, including cytoskeleton formation, cell movement, and intra-cellular trafficking. During cell division, microtubules have increasingly critical functions that ensure proper chromosome segregation beginning in interphase and culminating in mitosis. The various functions of microtubules, including both cellular structure and movement, are possible due to their dynamic, hollow, and cylindrical polymeric structure. As polymers, canonical microtubules are formed from 13 protofilament chains composed of both alpha and beta tubulin dimers. This is an evolutionarily conserved structural feature present in all eukaryotic supergroups with the 13 protofilaments providing a linear lattice that runs parallel to the length of the polymer and may allow for direct transport along the microtubule [4]. The sequential addition of tubulin heterodimer (αβ/αβ/αβ/αβ…) creates a polarity within both the protofilament and entire microtubule. The termini of the microtubule are therefore referred to as either the minus end or plus end with alpha tubulin always capping the minus end and the beta tubulin capping the plus end. (Figure 1). The minus and plus termini do not function equally, as the plus termini lengthens and shortens at a faster rate than the minus termini [1,5]. This dynamic instability of the microtubule poles, the constant lengthening and shortening, is called microtubule dynamics. Microtubule dynamics allows the overall tubular structure of the microtubule to be utilized by the cell in of diverse set of functions, including mitotic chromosomal segregation and intracellular trafficking. Although variable, when viewed across the cell cycle, a regular pattern of microtubule dynamics emerges with a maximum occurring during mitosis.

The essential unit of both the protofilament and microtubule is the protein tubulin. There are six different eukaryotic families (Alpha, Beta, Gamma, Delta, Epsilon, and Zeta) that comprise the tubulin superfamily. The alpha and beta tubulin families are the major constituents and serve as the primary components that form the microtubule. The gamma tubulin family forms gamma tubulin ring complexes (gamma-TuRC) that serve as templates for microtubule nucleation [6]. The other three tubulin families have specialized functions and are typically associated with the centriole [7]. Due to their central role, both alpha and beta tubulin have multiple isotypes. The alpha tubulin family consists of eight isotypes, while the beta tubulin family has nine isotypes. Members of the beta family share immense sequence and structural homology with primary sequence variation of only 4-16%, which is clustered at the C terminus [8,9,10]. This C-terminal variability is the basis for beta tubulin isotope identification and naming convention [9]. Beta tubulin isotype expression can be either constitutive or highly tissue specific. For example, beta tubulin isotope IVa typically has high expression in neurons, while isotype I is found in almost all cell types [8]. However, the diversity of both alpha and beta tubulin isotypes does not limit their incorporation within the same microtubule. Due to this observation, microtubules have been called “mosaics” that are formed from the variety of tubulin isotype populations that exist within the cellular milieu [11]. Characterization of the effect that tubulin isotype diversity has on its organization or patterning within the microtubule and how this impacts both microtubule function and MTA treatment is ongoing [11,12]. Importantly, while both alpha and beta tubulins have similar masses and three dimensional structures, all MTAs bind exclusively to members of the beta tubulin family.

Alpha and beta tubulin exist in an equilibrium between a soluble pool of tubulin heterodimers and polymerized tubulin that is assembled into microtubules [11]. This equilibrium changes throughout the cell cycle, where the microtubule half time varies from minutes to hours during interphase but only 10–30 s in mitosis [13]. This peak of microtubule dynamics that occurs during mitosis is necessary for mitotic spindle formation. Correct formation of the mitotic spindle is tightly regulated and oversight of its proper formation is termed the spindle assembly checkpoint (SAC). Regulated spindle assemble and SAC progression are needed for successful chromosomal segregation [14]. The purpose of the SAC is to prevent improper chromosomal segregation through inhibition of the anaphase promoting complex (APC) [15,16]. The APC is an E3 ubiquitin ligase that targets cyclin B1 for degradation and serves as a primary signal for mitotic exit [15]. Cellular treatment with MTAs disrupts necessary microtubule dynamics during mitosis which results in an increase in SAC inhibition of the APC and ultimately SAC dependent mitotic arrest [15]. Physiologically, this type of arrest is necessary to allow all kinetochores located at chromosomal centromeres time to organize and attach to the polar microtubules that will guide sister chromatids to opposite poles of mitotic cells [14,17]. The physiologic increase of microtubule dynamics necessary for mitotic spindle formation serves as a viable chemotherapeutic target due to the robust growth potential of cancer that increases the overall percentage of time that a cell spends in mitosis. MTAs interfere with this process and significantly prolong mitotic arrest inducing initiation of the desired apoptotic cascade.

## 3. Microtubule Targeting Agents (MTAs)

### 3.1. Background

MTAs were first identified in the middle of the 20th century through natural products research searching for anti-cancer agents [18]. Since their identification, both vinca alkaloids and taxanes have been used extensively as broad anti-cancer chemotherapeutics [19]. Initially, MTAs were discovered through their anti-mitotic ability, even before their effects on microtubule dynamics were understood. MTAs have been found to associate with one of four possible binding sites on beta tubulins [3]. Importantly, the site that a MTA binds to determines which of two possible outcomes the MTA induces on the microtubule. Association with either the colchicine or vinca alkaloid site results in microtubule depolymerization, while association with the laulimalide site or taxane/epithilone site stabilizes the microtubule [20]. Despite the advent of “second generation” anti-mitotic drugs developed to target specific proteins involved in chromosomal segregation, spindle formation, or mitosis [21], the initially identified microtubule targeting agents remain the chemotherapeutic gold standard, as many of these new compounds fail to retain sufficient cell killing potential [22]. For the purpose of this review, we will focus on the compounds that target either the vinca alkaloid (depolymerizing) or taxane (stabilization) sites due to their clinical relevance in cancer. For the vinca alkaloids, these include vinblastine (Velban) and vincristine (Oncovin), while for the taxanes, these include both paclitaxel (Abraxane) and docetaxel (Taxotere).

MTAs are a diverse set of compounds, while the primary function of taxanes and vinca alkaloids as either stabilizing or destabilizing divides them into two categories [1]. Interestingly, the biochemical outcome of either category of drug is shared at low concentrations, as they both cause an overall inhibition in microtubule dynamics [1]. This negative effect on microtubule dynamics has a direct effect on spindle efficacy that results in extended mitotic arrest [23]. This induces the cell to undergo programmed cell death (apoptosis), the clinically desired outcome of MTAs.

### 3.2. Vinca Alkaloids

Both of the vinca alkaloids that ultimately entered the clinic were discovered in the 1950s through analysis of extracts of the subtropical flower *Vinca rosea* (Catharanthus roseus) [24]. Vincristine was the first of these to be approved by the FDA in 1963, with vinblastine shortly following in 1965 [25]. Three other vinca alkaloids, vinorelbine, vindesine, and vinflunine, are semisynthetic derivatives of vinblastine, though they have not progressed to the clinic [3]. Although vincristine has neurotoxic effects, it is indicated for use in leukemias and lymphomas as well as lung and testicular solid cancers. Vinblastine, while myelosuppressive, is tolerated at higher doses than vincristine, and indicated for Hodgkin’s lymphoma, bladder, breast, and brain cancer [26]. Vinca alkaloids associate with both free beta tubulin and with exposed polymerized beta tubulin at the plus end of the microtubules. Mechanistically, vinca alkaloids function by binding to and sequestering free beta tubulin which limits its supply for microtubule extension. Likewise, vinca alkaloids can bind to polymerized beta tubulin, blocking further microtubule extension [27]. In each case, the compounds stop microtubule growth which switches the dynamics to favor loss in microtubule length and thus destabilization.

### 3.3. Taxanes

Like vinca alkaloids, paclitaxel was discovered in the early 1960s in extracts of *Taxus brevifolia* (Pacific Yew) bark during a National Cancer Institute screen for natural products with anti-cancer activity [18,28]. Subsequent characterization of paclitaxel’s chemical structure and its function on microtubules was completed over the next two decades [19,29,30]. Paclitaxel was initially only available as a natural product and with a limited supply of Pacific Yew a new source or synthetic scheme to make paclitaxel was sought. Docetaxel is a semi-synthetic derivative of a taxane, 10-deacetylbaccatin III, identified in *Taxus baccata* (English Yew) that was discovered as a result of the immense demand for paclitaxel [18]. A natural product from the English Yew, 10-deacetylbaccatin III, was a viable candidate to serve as an alternative starting point for the synthesis of paclitaxel. However, during the organic synthesis, an esterification reaction did not initially work which serendipitously directed the reaction to the end product of docetaxel [18]. Conveniently, docetaxel is more soluble than paclitaxel and exhibits less neurotoxicity [18,31]. Paclitaxel is indicated for multiple solid tumors including ovarian, breast, and lung, while docetaxel is indicated for breast, gastric, head and neck, and prostate cancers [24].

Unlike vinca alkaloids, paclitaxel and other taxanes predominantly bind polymerized beta tubulin at a separate site within the lumen of the microtubule nestled between parallel protofilaments [32]. Instead of destabilizing, this association stabilizes the protofilaments as well as the microtubule as a whole, inhibiting disassembly of the microtubule (also called microtubule catastrophe). As hydrophobic cytotoxic chemicals, MTAs are not without their problems, which include off-target toxicity and poor drug solubility. To this end, both vinca alkaloid and paclitaxel have served as the chemical foundation for larger chemical families that are derived from their chemical scaffolds. While these retain their specific regional interactions on beta tubulin (Figure 1) and their functional inhibition of microtubule dynamics, the majority of these novel chemicals do not retain the potency observed their parental compounds [31].

### 3.4. MTA Chemoresistance

Cancer cells have evolved to evade normal regulation of growth and differentiation. Many chemotherapeutics have been identified due to their ability to exploit such changes in cancer cell growth and metabolism. Yet, the same alterations that enable cancer to emerge leads to the proliferation of chemotherapeutic resistant populations that develops either the ability to evade chemotherapy induced death or the ability to remove the chemotherapeutic agents from the cellular environment. As taxanes and vinca alkaloids closely mimic one another’s cellular function, likewise, many of the same mechanisms are employed to gain resistance to both vinca alkaloids and taxanes. These include the induction of multi-drug resistance efflux transporters, e.g., P-glycoprotein, that pump hydrophobic natural products, including vinca alkaloids and taxanes, out of the cell [28]. Interestingly, as low concentrations of both of these two classes of MTAs were shown to perturb microtubule dynamics, it was proposed that this mechanism might not be as detrimental to MTA activity as once thought [1]. This may explain why the co-treatment of MTAs with transport inhibitors has seen limited success [33,34]. Another mechanism of MTA resistance is the diversity of tubulin isotypes. There are nine different isotypes of beta tubulin located on separate genes and with different primary amino acid sequences. It was shown that some beta isotypes are more resistant to MTA treatment. Specifically, the beta tubulin III isotype is particularly troublesome for both paclitaxel and vinca alkaloids [8,33,35]. Typically, beta tubulin III has been shown to increase microtubule dynamics and is physiologically observed in neurons where it plays a role in cellular projections or neurites [36]. When elevated in cancer cells, this modulation of microtubule dynamics appears to destabilize the microtubule and mitigate MTA efficacy [8,36]. A third mechanism of MTA chemotherapeutic resistance is suppression of the apoptotic response. A primary function of MTA treatment is the induction of apoptosis or programmed cell death. It was shown that MTA treatment increases post-translational modification of the anti-apoptotic BCL2 family through kinase activation (JNK, CDK1, and many others [37,38,39]. Although phosphorylation of these anti-apoptotic proteins can either facilitate or inhibit their function, as a whole, this type of phosphorylation event is primarily inhibitory and, thus, pro-apoptotic [3,40]. Yet, efficacy of MTA compounds can be attenuated through presence or upregulation of anti-apoptotic proteins [41]. Indeed, this was shown to occur through the altered expression of proteins, such as p53 and BCL2 [28,42]. Importantly, it was shown that the MTA drug concentrations needed to directly promote apoptosis are not met in vivo [43]. This necessitates combination therapy of MTAs with other chemotherapeutic or targeted approaches. Functional MTAs canonically induce cell death during mitosis, with intrinsic apoptosis serving as the major mechanism regulating mitotic cell death [44,45]. In human cell, the intrinsic apoptosis response is primarily regulated by the BCL2 family. Thus, MTA efficacy and BCL2 functionality are intrinsically linked and this link has been strengthened through targeted studies over the past two decades [46]. Likewise, MTA resistance was shown to be strongly impacted by BCL2 family expression and function [47,48]. These links between the BCL2 family and both MTA efficacy and resistance uniquely position the BCL2 family to be targeted alongside MTA therapy.

## 4. BCL2 Family of Proteins

To understand the impact of the BCL2 family on MTA efficacy, it is first necessary to review how the BCL2 family regulates apoptosis. BCL2, the eponym of the BCL2 family, was initially observed in the late 1970s through its upregulation due to chromosome 14 translocations in lymphomas [49,50]. Within the next decade, human BCL2 was cloned and identified as an anti-apoptotic oncogene with the potency to protect against multiple forms of cellular stress [50,51,52]. Subsequent sequence analysis has identified a number of proteins that share from one to four homologous sequence motifs, referred to as BCL2 homology (BH) motifs with BCL2, thus making them part of the BCL2 family (outlined in Reference [53]). Based on the composition of these four motifs (BH1, BH2, BH3, and BH4), the BCL2 family can be divided into three main categories: Anti-apoptotic, pro-apoptotic effectors, and pro-apoptotic activators. As a group, the BCL2 family is a collection of both pro- and anti-apoptotic proteins that are evolutionarily conserved and found in all animals [54]. The function of the BCL2 family is diverse, extending beyond apoptosis with important roles in both development [55] and homeostasis [56]. The dysregulation of the BCL2 family, either through the upregulation of anti-apoptotic members or downregulation of pro-apoptotic members, has been implicated throughout tumorigenesis and tumor progression [57]. The interplay between the pro-survival and pro-apoptotic BCL2 proteins provides the mechanism or biochemical balance that cellular signaling is filtered through in order to determine cell fate. In the event of increased pro-apoptotic signaling, the BCL2 family can initiate a cascade that concludes with caspase activation, cellular degradation, and phagocytosis in a manner that does not stress the neighboring tissue [58,59].

**Figure 2:**
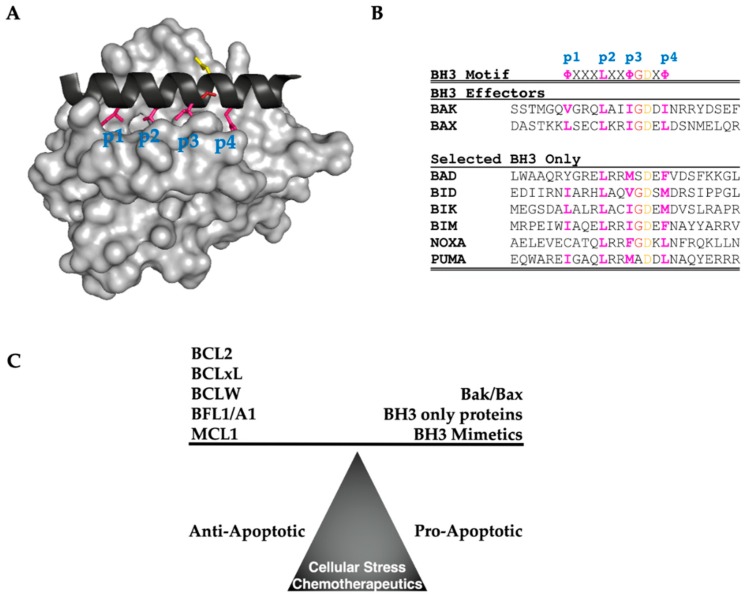
BCL2 Family Structure, Function, and Equilibrium. (**A**) Surface structure of MCL1 (grey) with BH3 binding pocket, representative of the anti-apoptotic BCL2 family, bound to BIM BH3 Only protein (Black) with highlighted residues: Hydrophobic (Pink), conserved Glycine (red), and invariant Aspartic Acid (Yellow). [PDB: 2NL9] (**B**) BH3 Sequence homology between BCL2 effectors and selected BH3 Only proteins. (**C**) BCL2 Family Interactions Balance Cell Stress Signaling, where BCL2 family stoichiometry and affinity dictate cell fate.

The focal point of BCL2 family regulation centers on the pro-apoptotic effectors, BAK and BAX, which can be induced through upregulation or activation to form homo-oligomers in the mitochondrial outer membrane. These oligomers form pores in the mitochondrial outer membrane, inducing mitochondrial outer membrane permeabilization (MOMP), a necessary event for cytochrome c release and subsequent apoptosis. In a non-stressed cell with basal expression of BAK and BAX, these proteins reside in the cytosol or mitochondrial outer membrane, respectively, in a monomeric and inactive state. Stress-induced upregulation of either BAK or BAX or one of the BH3-only activators (BID, BIM, or PUMA), can induce BAK or BAX oligomerization. In either case, oligomerization of BAK and BAX can be held in check by the presence of anti-apoptotic BCL2 family members (BCL2, BCLxL, BCLW, MCL1, and BFL1/A1) that contain all four BH motifs structurally folded in such a way to form a hydrophobic groove or pocket. This pocket binds to amphipathic alpha helical BH3 motifs that are present in both classes of pro-apoptotic BCL2 family members. The affinity of the BH3 helix and the BH3 binding pocket is the hinge between the two poles, pro-apoptotic and pro-survival, within the BCL2 family and the basis for BCL2 regulation of cell fate. The canonical BH3 helix is a structural motif whose primary sequence is capped in the C-terminal direction with aspartic acid [53]. Moving in the N-terminal direction, the next residues are often glycine, followed by isoleucine. The rest of the BH3 primary sequence is interspersed with both charged and polar residues, such that when the BH3 helix is folded, the hydrophobic residues reside throughout one half of the long axis of the helix, thus creating its amphipathic nature. The four hydrophobic residues of the BH3 bind into specific pockets (p1-p4) [60,61,62] within the BH3 binding groove (Figure 2). This hydrophobic side of the BH3 helix is largely the driver of binding to the hydrophobic BH3 pocket found on the anti-apoptotic BCL2 family members.

Association of anti-apoptotic BCL2 family proteins with either the BAK or BAX BH3 helix inhibits their oligomerization. The third BCL2 subfamily, the BH3 only proteins, retain only the BH3 motif. Two prevailing models (direct and indirect) were proposed to describe the impact that BH3-only proteins utilize to regulate the BCL2 family [53,63]. In the direct model, BH3 only proteins bind directly to the pro-apoptotic effectors, BAK and BAK, stimulating oligomerization [64,65,66]. In the indirect, BH3 only protein act as apoptotic sensitizers, inhibiting the anti-apoptotic BCL2 family members and thereby allowing BAK and BAX to oligomerize [63,67,68]. The BH3-only subfamily contains over 20 identified members in humans, and different members were shown to favor one or the other of these models. Further, it was demonstrated that in the absence of BH3 only proteins, BAK and BAX, are able to homo-oligomerize and permeabilize the mitochondrial outer membrane [69]. Thus, both the direct and indirect activation of pro-apoptotic effectors are not mutually exclusive and a unified theory that combines both was proposed [63]. Not unlike tubulin heterodimers cycling between polymerized tubulin within the microtubule and solubilized tubulin populations, BAK and BAX are also able to translocate between the cytosol, endoplasmic reticulum, and the mitochondrial outer membrane [70,71]. Regulation of this process mimics the general regulation of BAK and BAX oligomerization with BAK and BAX retro-translocation away from the mitochondria being carried out by the anti-apoptotic BCL2 family members [72], while the BH3 only protein, BIM, is involved in BCL2 translocation back to the mitochondria [66]. Thus, the BCL2 family regulates not only BAK and BAX oligomerization, but also their localization into the mitochondrial outer membrane in their regulation of stress-induced apoptosis.

### 4.1. Anti-Apoptotic BCL2 Family and MTAs

The BCL2 family serve as the gatekeepers of an irreversible intrinsic apoptotic cascade. As critical regulators of cellular viability, the BCL2 family effectively determines cell fate through the interpretation and status of cellular stress. For this reason, many of the functions and capabilities of the BCL2 family have been elucidated through their effects on chemotherapy [41]. The treatment of cancer cells goes beyond the natural stress that tumorigenesis can often induce (e.g., chromosomal abnormalities, metabolic stress), as broad-spectrum chemotherapeutics intentionally induce acute cellular stress. MTAs targeting of microtubule dynamics introduces enormous stress on the cell to which the anti-apoptotic BCL2 family members are obligated to respond to enable the cell to survive. Repeatedly, studies have highlighted the importance of the BCL2 family in mitigating the effects of MTAs, both taxanes [21] and vinca alkaloids [3]. Interestingly, both of these drug classes have been shown to collectively initiate downstream effects on all five anti-apoptotic BCL2 family proteins. Given the importance of the anti-apoptotic BCL2 family members in enabling cancer cell survival, a number of small molecule inhibitors of this family were identified and are being tested for their anti-cancer activity. With the first of these compounds entering the clinic [73], one interesting question to be answered is how the combination of MTAs with different and individually specific anti-apoptotic BCL2 inhibitors can overcome MTA resistance or improve response. Thus far, a number of studies investigated cell lines and xenograft models to determine the impact that anti-apoptotic protein expression or suppression has on MTA activity. These studies are reviewed in the following sections and summarized in Table 1.

### 4.2. BCL2

As the namesake of the BCL2 family and the template anti-apoptotic family member, the BCL2 protein plays an unexpected role in MTA sensitivity. As an anti-apoptotic protein, it might be expected that elevated levels of BCL2 protein expression would lead to MTA resistance. Interestingly, the opposite is observed in both lung and breast cancer lines where elevated BCL2 protein expression has been shown to increase paclitaxel sensitivity [37]. This is supported by the observation in prostate cancer models that loss of BCL2, such as in the BCL2 null cell line (DU145), corresponds with resistance to paclitaxel, while a BCL2 expressing cell line (PC3) is sensitive to paclitaxel [39]. One possible explanation for this effect is that elevated BCL2 expression coincides with increased expression of the pro-apoptotic, BH3 only protein, BIM [37]. Thus, when the cell overexpresses BCL2 to try and evade death, the corresponding upregulation of BIM balances the scales to ultimately provide no protective effect. In addition, a number of studies suggest that paclitaxel can be sequestered directly by BCL2 [85,86,87] and that this binding inhibits the pro-apoptotic effect of paclitaxel. As it was shown that the BCL2 protein is known to associate with microtubules [88], this may increase the stochastic possibility of BCL2 binding to and sequestering paclitaxel. In further agreement, loss of BCL2 was also associated with resistance to the vinca alkaloid, vinflunine, in ovarian cancer cells [89]. These data suggest that in solid tumors, BCL2 expression actually sensitizes cancer cells to the apoptotic inducing effect of MTAs. Intriguingly, the opposite effect is seen in the 697 leukemia cell line where overexpression of BCL2 protects these from paclitaxel-induced apoptosis [74]. This alternative effect similarly observed in another leukemia cell line (HL-60) wherein extended treatment with both paclitaxel and vincristine have been shown to decrease BCL2 mRNA expression [75]. These studies demonstrate that cell type, particularly the difference between solid and hematopoietic tumors, can impact both the presence and role of both BCL2 protein in its regulation of MTA treatment sensitivity.

### 4.3. BCLxL

Although it shares many functional similarities with its anti-apoptotic homolog, BCL2, the anti-apoptotic BCL2 family member, BCLxL, does not have as complicated a relationship with MTAs as BCL2. Exogenous expression of BCLxL was shown to protect both leukemia and solid tumor cells from paclitaxel-induced cell death [76,90]. Further, while targeted inhibition of BCLxL using a BH3 mimetic alone had minimal effect on cell viability, combination of this compound with paclitaxel led to a synergistic response [90]. The ability for BCLxL inhibitor to work in combination with taxanes was further demonstrated in a study to evaluate how selective and targeted inhibition of BCLxL impacts docetaxel treatment of an array of mouse xenografts [79]. These studies highlight the potential of using anti-apoptotic BCL2 family inhibitors alongside MTAs to improve therapeutic response.

### 4.4. BCLW and BFL1/A1

Both of the anti-apoptotic BCL2 family members BCLW and BFL1/A1 function as much as BCLxL with regard to their impact on MTA sensitivity. Analysis of siRNA knockdown or CRISPR-Cas9 deletion of BCLW resulted in an increase in the rate of paclitaxel-induced cell death [77], while overexpression of BCLW prolonged cell viability in the presence of paclitaxel [77]. Further, exogenous upregulation of BFL1/A1 in leukemia cells decreased apoptotic induction by paclitaxel [78]. This impact on MTA activity was also observed in vinca alkaloid combinations in a murine lymphoma model. In these studies, cellular resistance to the vinca alkaloid, vinfuline, was characterized by elevated BFL1/A1 protein levels [84]. These studies highlight the necessity of targeting the specific anti-apoptotic BCL2 proteins that seem to be upregulated in a compensatory manner upon MTA treatment.

### 4.5. MCL1

The final anti-apoptotic BCL2 family member, MCL1, has been closely linked with MTA resistance. This involvement in MTA chemoresistance assisted in identifying MCL1′s key role in acting as a mitotic clock and serving as the key anti-apoptotic BCL2 protein during mitosis [44]. During extended mitotic arrest, the cell is presented with two options: Death or mitotic slippage. Throughout this cellular decision, the abundance of MCL1 protein was identified to be a critical factor that acts as a mitotic timer [48,91,92]. When MCL1 protein levels are low or absent, the arrested cell is tipped into the apoptotic cascade. But, when MCL1 is in high abundance, cellular viability is maintained and the cell may either correct itself and divide with proper chromosomal separation or experience mitotic slippage. When the cell escapes mitotic arrest through mitotic slippage, the cell bypasses the final stages of mitosis, which often results in aberrant cytokinesis, including incomplete or asymmetric chromosomal segregation. This ultimately leads to the presence of abnormal chromosome copy numbers in daughter cells. Clinically, mitotic slippage caused by prolonged MTA-induced mitotic arrest is undesirable. Since MCL1 was recognized as regulator of mitotic escape, a number of studies continue to highlight how modulation of MCL1 effects MTA treatment [80,81,93].

Initial studies to assess the impact of MCL1 on MTA sensitivity highlighted the impact that MCL1 levels have on both sensitivity and resistance to taxanes and vinca alkaloids [48]. Likewise, knockdown of PIAS1, an E3 SUMO ligase that is linked with decreased MCL1 protein levels, was shown to sensitize a docetaxel-resistance PC3 sub-cell line to docetaxel [80]. Regulation of MCL1 expression can similarly impact MTA activity as it has been shown that expression of the RNA binding protein, PTBP1 (HUR1) regulates sensitivity to both vincristine and paclitaxel through its regulation of MCL1 [81]. Interestingly, the vinca alkaloid, vinblastine, is able to mediate a decrease in MCL1 protein through phosphorylation events leading to its ubiquitination [82,83]. Multiple drugs have been developed to inhibit the BCL2 family, while it is notable that cancer sensitivity to combination therapies that include MTAs can be adversely affected by drug regulation of the BCL2 family. For instance, therapeutic combination of proteasome inhibitors, such as Bortezomib, and MTAs are employed in the clinic to treat multiple cancers [94]. Yet, proteasome inhibition was shown to increase MCL1 protein levels (as it blocks MCL1 proteolytic degradation). This increase was shown to inhibit taxane-induced mitotic death [91].

## 5. Combination of BCL2 Family Inhibitors and MTAs

The tension between the pro- and anti- apoptotic BCL2 proteins dictated by both stoichiometry and binding affinity results in a homeostatic balance within the BCL2 family (Figure 2). Cancers that are addicted to the anti-apoptotic side of the family [41] are tipped toward the pro-survival direction. One method to therapeutically correct this scale is to introduce BH3 mimetics, small molecules that bind into the hydrophobic BH3 pocket of the anti-apoptotic members, and displace the pro-apoptotic activators and effectors. These inhibitors were first developed to bind to BCLxL and BCL2 and utilized the p3 and p4 hydrophobic pockets, as well as mimicking the conserved aspartate salt bridge (Figure 2). Thus far, a specific inhibitor for BCL2 (ABT-199, Venetoclax [95]) obtained approval for treatment of chronic lymphocytic leukemia (CLL). However, leading up to the FDA approval of ABT-199, many other small molecules inhibitors of the BCL2 family were developed, and these have been invaluable for pre-clinical assessment of anti-apoptotic BCL2 targeting. These include, ABT-737 [96], ABT-263 (Navitoclax) [97], Sabutoclax [98], A1210477 [79], and S63845 [41,99]. The utility of these individual small molecule inhibitors is that while they are all BH3 mimetics, they do not bind equally to all of the anti-apoptotic BCL2 family members. The first of these inhibitors to be developed, ABT-737, specifically targets BCL2, BCLW, and BCLxL as is the clinical candidate that was developed based on its initial preclinical success, ABT-263. Sabutoclax was the first pan-active BCL2 inhibitor capable of targeting all 5 anti-apoptotic proteins. A1210477 and S63845 followed up on the promise that MCL1 inhibition seemed to offer as MCL1 specific inhibitors. This has allowed direct studies to determine the importance of individual BCL2 proteins both as single agents and in combination with other chemotherapeutic agents.

**Figure 3:**
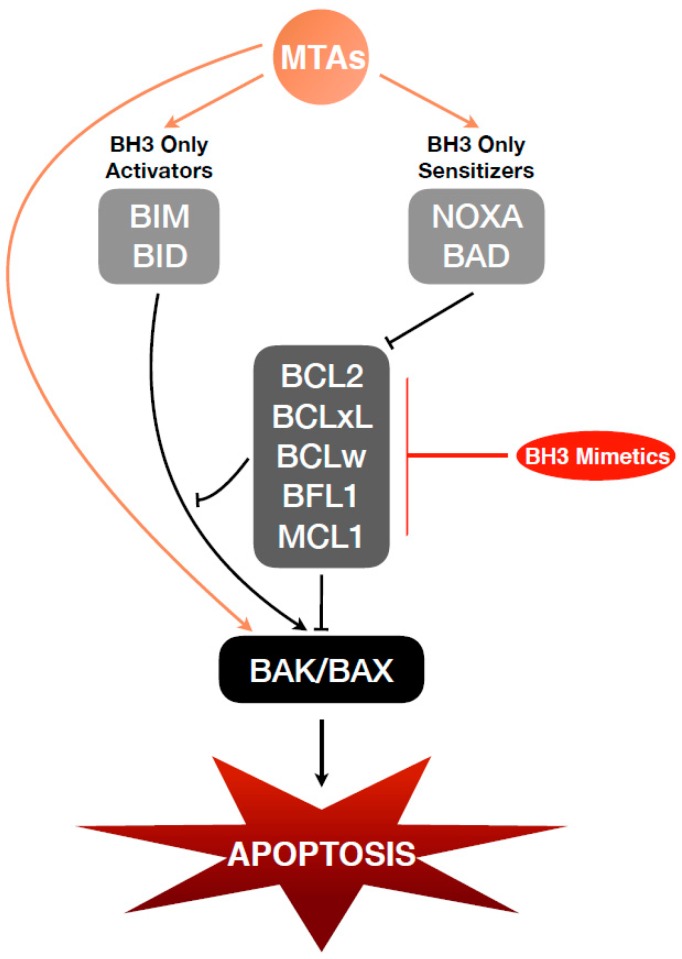
Mechanism of MTAs and BH3 Mimetics on BCL2 Family Signaling. MTAs induce cellular stress that leads to upregulation of pro-apoptotic BCL2 family proteins and ultimately apoptosis. Cancer cells can overcome this through upregulation of anti-apoptotic BCL2 family proteins. BH3 mimetics directly target the anti-apoptotic BCL2 family members to inhibit their suppression of BH3-only activators and/or of BAK/BAX oligomerization.

The identification that anti-apoptotic BCL2 family members can regulate cellular sensitivity to MTAs led to a series of studies evaluating how these various inhibitors combine with both taxanes and vinca alkaloids. Thus far, each of these compounds been shown to increase the efficacy of MTAs (Table 2). Figure 3 demonstrates how BH3 mimetics feed into BCL2 signaling resulting in cell death. The first developed and tested BH3 mimetic, ABT-737, was shown to sensitized multiple different cancer cell types (breast [100], prostate [101], melanoma [102], and hepatoblastoma [103]) to taxanes. MCL1 inhibition further provided synergistic activity with docetaxel in both prostate cancer cell lines and xenograft studies [101]. It is perhaps no accident that ABT-199, specific for BCL2, was approved for treatment of CLL based on the differences observed in MTA regulation of BCL2 between solid and liquid tumors. This effect is recapitulated by small molecule inhibition of BCL2, where in both prostate and breast cell models ABT-199 and BCL2 inhibition do not synergize with paclitaxel treatment but both ABT-737 [104] and ABT-263 [105] do synergize, likely due to their inhibition of BCLxL. In prostate cells, inhibition of BCLxL has been shown to be beneficial, though not equally, for both paclitaxel sensitive and resistant cells [105]. Teasing apart the effects of BH3 mimetics that target more than one BCL2 protein has led to the continued development of inhibitors for individual anti-apoptotic BCL2 members. Inhibitors of both MCL1 and BCLxL are in clinical trials [73,106,107]. Unlike BCL2 inhibition, the MCL1 specific inhibitor, S63845, has shown promise in targeting and sensitizing triple negative breast cancer to docetaxel [108]. From these early studies, BH3 mimetics may enable significant reduction in MTA dosing while retaining clinical effect. As more potent and specific anti-apoptotic BCL2 family inhibitors emerge, continued assessment of their impact on MTA sensitivity will be important not only to identify cancers that can be best treated through combination of BH3 mimetics and MTAs, but also as BH3 mimetics may resensitize cancers to MTA treatment.

## 6. Conclusions

Since their identification, MTAs have become the standard of care in treating many cancer malignancies. However, MTAs are not without their problems, namely toxicity toward healthy tissues, including the brain. The BCL2 family, as the key regulators of programmed cell death and crucial mediators for cellular death during mitosis, play a key role in MTA effectiveness. The recent development of small molecules that can specifically target the BCL2 family opened a new avenue for improving cancer therapy and, specifically, MTA effectiveness. The clinical potential of combining MTAs with BCL2 inhibition is only beginning to be explored, yet this limited work already hints at promising new combinations that exploit the ability of BCL2-inhibitors and MTAs to work in synergy to improve targeted cancer cell death, allow for reduced MTA dosage, and result in decreased toxicity. However, knowing when and where to target the BCL2 family individually or as a whole will lead to better combinatorial therapies with existing MTA therapies.

## Figures and Tables

**Table 1:** MTA Efficacy and sensitivity within the context of the anti-apoptotic BCL2 proteins.

MTA	Protein	Cell Type/Model	Effect	Reference
Paclitaxel	BCL2	Breast, Lung, Prostate	* Presence sensitizes	[37,39]
	BCL2	Leukemia	Presence induces resistance; paclitaxel decreases BCL2 mRNA expression	[74,75]
	BCLxL	Leukemia, Colon	Upregulation induces resistance, inhibition sensitizes	[76]
	BCLW	Leukemia	Knockdown/out sensitizes	[77]
	BFL1/A1	Leukemia	Upregulation induces resistance	[78]
Docetaxel	BCLxL	Lung, Myeloma	Inhibition sensitizes	[79]
	MCL1	Prostate	* Inhibition or downregulation sensitizes	[80,81]
Vincristine	BCL2	Leukemia	Treatment decreases BCL2 mRNA expression	[75]
Vinblastine	MCL1	HeLa	Treatment decreases MCL1 protein levels	[82,83]
Vinflunine	BFL1/A1	Lymphoma	Increase linked with resistance	[84]

* Denotes studies that were performed in both cell lines and mouse xenograft models.

**Table 2:** BCL2 family inhibitors and their effect in combination with MTAs.

BH3 Mimetic	MCL1	BFL1/A1	BCLW	BCLxL	BCL2	Cell Type/Model	Effect	Reference
**ABT-737**			X	X	X	Melanoma, Breast, Prostate, Liver	Sensitizes cells to paclitaxel or docetaxel	[100,102,103]
**ABT-263**			X	X	X	Prostate	Additive with vincristine; synergy with paclitaxel	[104,109]
**ABT-199**					X	Leukemia, Lymphoma	Approved for CLL; sensitizes cells to paclitaxel	[95,110]
**WEHI-539**				X		Colon	Sensitizes to Paclitaxel	[90]
**Sabutoclax**	X	X	X	X	X	Prostate	Sensitizes to docetaxel	[81,93]
**AT-101**	X		X	X	X	Breast	Synergizes with paclitaxel	[111,112]
**S63845**	X					Breast	Synergizes with docetaxel	[107,108]
**A1210477**	X							[79]
**AMG-176**	X							[113]
**AZD-5991**	X							[114]
